# Genetic variation and population structure in China summer maize germplasm

**DOI:** 10.1038/s41598-021-84732-6

**Published:** 2021-04-13

**Authors:** Guoping Shu, Gangqiang Cao, Niannian Li, Aifang Wang, Fang Wei, Ting Li, Li Yi, Yunbi Xu, Yibo Wang

**Affiliations:** 1 Center of Biotechnology, Beijing Lantron Seed, Zhengzhou, 450001 Henan China; 2 School of Agricultural Science, Zhengzhou University, Zhengzhou, 450001 Henan China; 3 Institute of Crop Science, National Key Facility of Crop Gene Resources and Genetic Improvement,Chinese Academy of Agricultural Science, Beijing, 100081 China; 4 Zhengzhou University Graduate Student Training Base at Beijing Lantron Seed, Zhengzhou, 450001 China; 5 Henan LongPing-Lantron AgriScience & Technology Co., LTD, Zhengzhou, 450001 Henan China

**Keywords:** Genetics, Plant sciences

## Abstract

Maize (*Zea mays* L.) germplasm in China Summer maize ecological region (CSM) or central corn-belt of China is diverse but has not been systematically characterized at molecular level. In this study, genetic variation, genome diversity, linkage disequilibrium patterns, population structure, and characteristics of different heterotic groups were studied using 525,141 SNPs obtained by Genotyping-By-Sequencing (GBS) for 490 inbred lines collected from researchers at CSM region. The SNP density is lower near centromere, but higher near telomere region of maize chromosome, the degree of linkage disequilibrium (r^2^) vary at different chromosome regions. Majority of the inbred lines (66.05%) show pairwise relative kinship near zero, indicating a large genetic diversity in the CSM breeding germplasm. Using 4849 tagSNPs derived from 3618 haplotype blocks, the 490 inbred lines were delineated into 3 supergroups, 6 groups, and 10 subgroups using ADMIXTURE software. A procedure of assigning inbred lines into heterotic groups using genomic data and tag-SNPs was developed and validated. Genome differentiation among different subgroups measured by F_st_, and the genetic diversity within each subgroup measured by GD are both large. The share of heterotic groups that have significant North American germplasm contribution: P, SS, IDT, and X, accounts about 54% of the CSM breeding germplasm collection and has increased significantly in the last two decades. Two predominant types of heterotic pattern in CSM region are: M-Reid group × TSPT group, and X subgroup × Local subgroups.

## Introduction

Maize (*Zea mays L.*) is the largest crop by acreage in China and it is an important source of food, feed, and industrial material in China. China Summer Maize (CSM) ecological region, also called China central corn belt, or Huanghuai Corn belt, including almost entire area of Henan, Shandong, Shaanxi Province, Southern part of Hebei, Shanxi Province, and Northern part of Jiangsu and Anhui Province, is the second largest among 6 ecological regions in China. The CSM region with more than 14 million hectare accounts for 35% of the national corn planting acreages and more than 40% of grain corn output in China.

The ecological environment and cultivation and crop rotation system in CSM ecological area are unique in China and in the world. Farmers grow two crops per year. The corn growing at CSM region is called summer maize because it was seeded in early summer, mostly late May and June, right after harvesting wheat and canola. The typical weather pattern at CSM region are: high soil temperature at corn seeding time, drought and strong wind in the middle stage of corn vegetative growth, high temperature and heat in flowering and silking, and continuous raining and severe disease at late stage of grain filling and kernel moisture dry-down. The corn has to be harvested from the field to get the farmland ready for planning winter crops (mostly winter wheat and canola) timely in later September or early October. Because inbred lines and hybrids from North America and Europe do poorly at CSM region and are difficult to be directly used in hybrid creation and corn production, researchers have long faced the challenges of broadening the germplasm basis of breeding population.

Maize was introduced into China nearly 500 years ago. The local germplasm widely used at present days, such as, Tang SiPingTou (referred to as TSPT), and LvDa Red Cob (Referred to as LRC) originated from a government-sponsored national collection of open pollinated populations from local farmers in early 1950s and have accumulated a large number of unique genetic mutations well adapted to China spring corn region and summer corn region (CSM) as well^1,2^.

Starting from 1950s, maize germplasms from all parts of world, mostly from North America have been brought into CSM region multiple times for new traits and yield heterosis enhancement^1,3^.

Three big germplasm introductions to China in the last four decades that have significant impact on the formation of modern maize germplasm at CSM region are: (1) introduction of Pioneer hybrids P78599, P3147, and P3382 as breeding germplasm in the later 1980s to bring in disease resistance genes, that leads to the formation of P heterotic subgroup and M-Reid_PA subgroup (2) the introduction of over 300 ex-PVP inbreds into China in the last decade and (3) the successful commercialization of hybrid Xianyu 335 and other hybrids by Pioneer Hybrid International in China’s Spring corn region at early 2000s and their expansion to CSM region at 2010s. Both (2) and (3) have enhanced the fusion and integration of North American germplasm into Chinese germplasm and explain the existence of the heterotic subgroup IDT and subgroup X in CSM germplasm. Using these new germplasm and the breeding methodology of pyramiding breeding of favorite traits^4^, a large number of inbreds and commercial hybrids with better grain yield, low kernel moisture, fast dry-down, and suitability to mechanical harvesting have been created in the last two decades.

Maize is one of the most successful crops in heterosis utilization through commercial hybrids, in particular, single-cross hybrids. To increase the probability of obtain high hybrid vigor in F_1_ hybrids, researchers assign inbred parents into different germplasm groups, also different gene pools, called heterotic groups, and create F_1_ hybrids by inter-heterotic group hybridization in order to obtain superior F_1_ heterosis in yield and many other traits; they improve the traits of a parental inbred line itself by intragroup hybridization to avoid heterosis or non-additive effect and to obtain large genetic gain in trait selection. The heterotic groups in US breeding germplasm were well studied^5,6^, and the heterotic groups in Chinese breeding germplasm were also studied in detail^1–3,7–9^.

Traditionally a maize inbred line is assigned to a particular heterotic group based on the field experiment data (the Combining Ability Test) from crossing the inbred line with a set of testers (a set of representative inbred lines from one or more heterotic groups)^8,10,11^. In recent years, researchers start to assign an inbred line to a heterotic group using genome data, in particular, a large set of SNP marker data. Several studies on germplasm diversity and heterotic group pattern using large scale diversity panels of inbreds and SNP data have been reported on world-wide maize inbred collection^12–15^ and North American germplasm^14,16–18^. Several studies also have been reported on Chinese germplasm in general^15^, and germplasm with focus to a specific ecological region, such as Northern China Spring Maize^19^, Southwestern China Spring Maize^20,21^. However germplasm at China Summer Maize (CSM) region has not been studied systematically at molecular level, and many important aspects of CSM germplasm are poorly understood. In this study, 490 maize inbred lines collected by CSM region researchers were studied using 525,141 SNPs obtained by Genotyping-By-Sequencing (GBS), to address the following questions from breeders and geneticists as well: (1) the degree of genetic diversity and genome differentiation (2) the population structure and heterotic group identity (3) major heterotic groups and predominant heterotic pattern of commercial hybrid creation (4) historical changes in germplasm composition and heterotic group in CSM region .

## Materials and methods

### Plant materials, DNA sequencing, and data processing

A panel of 490 maize inbred lines was collected from researchers at China summer corn-belt, with origin from China Summer Corn (CSC, 209),North East China (NE China, 170), North West China (NW China, 1), South West China (SW China 32), and USA (US, 78). Leaf sample from each line was used for DNA extraction with a CTAB procedure. DNA sequencing follows a protocol of Elshire et al.^22^. Genomic DNA was digested with the restriction enzyme ApeK1. GBS libraries were constructed in 96-plex and sequenced on Illumina HiSeq 2000. SNP calling was performed using TASSEL-GBS pipeline^23^ with Maize B73 RefGen_v3 as the reference genome. Initially, 877,631 SNP loci went through a quality control procedure that filters out SNP loci with high missing rate and spurious heterzygocity arising from sequencing error and the artifactual SNPs originating from paralogous tags^14,23,24^; 876,305 of them were assigned to chromosomes 1 to10, and 1326 of them could not be anchored to any of the 10 maize chromosomes and were excluded. Then data was loaded into TASSEL 5.2^25^(https://www.maizegenetics.net/tassel), and SNP loci with minor allele frequency (MAF) < 5% / missing rate > 20% / residual heterozygocity > 5% were further filtered out, and data for 525,141 high-quality SNP loci was kept for all analyses involving the entire data set. For all subpopulation-specific analysis, tagSNP data instead of single locus SNP were used, loci with  missing rate > 10% or with residual heterozygocity were treated as missing and were excluded from calculation.

### SNP characteristics

Allele frequency analysis of both unfiltered and filtered data was carried out with TASSEL5.2 software. The Polymorphic Information Content (PIC)^26^ and Genetic Diversity (GD) were calculated ether using PowerMarker V 3.25^27^ or manually^28^. MAF and Heterozygosity (H) were calculated using TASSEL5.2. The pairwise relative kinship between two inbreds was estimated using 525,141 SNP loci by TASSEL 5.2.

### Linkage disequilibrium, haplotype and tagSNPs

A chromosome was divided into 50 kb segment and the pair-wise LD was calculated using Pearson correlation coefficient (${\mathrm{r}}^{2}$) by TASSEL5.2 and average LD was assessed for each segment. The distribution of LD along each chromosome was plotted by R package ggPlot2 (https://ggplot2.tidyverse.org/) . For the LD pattern of entire inbred collection (Table 1, Fig. 2c, Fig. S2), the entire set of 525,141 SNP loci from 490 inbred lines were used; for the LD pattern of a particular subgroup (that is, subgroup-specific LD, See Fig. 6), 4849 tagSNP loci, which represent 3618 haplotype blocks generated by Haploview V4.2 (https://www.broadinstitute.org/haploview/haploview) from the filtered SNP data were used.

**Table 1:** The LD, LD decay distance, LD block, and TagSNPs.

Chromosome	LD decay distance (Kb)	Mean r^2^	No. of LD blocks	No. of TagSNPs
1	80	0.126	533	708
2	55	0.125	448	594
3	170	0.129	411	554
4	120	0.125	478	644
5	115	0.132	321	427
6	65	0.124	279	381
7	72	0.126	291	397
8	35	0.123	304	405
9	245	0.133	260	356
10	240	0.137	293	383
Mean	165	0.128	361.8	484.9

### Population structure detection and characterization

ADMIXTURE 1.3.0 was used to detect the population structure among all 490 maize inbred lines using 4849 tagSNPs. Principal component analysis (PCA) based on 525,141 SNPs was performed using the R package SNPRelate. Pairwise Fixation Indexes (F_st_) from Wright^29,30^ was calculated using a software module of ADMIXTURE 1.3.0 (http://dalexander.github.io/admixture/index.html).

### Group identity assignment

The Q value, the probability of an inbred line belonging to a particular group or the genomic contribution from a group to the inbred line, was the output of ADMIXTURE 1.3.0. An inbred line would be assigned a group membership or Group ID by Default (see the column: Group ID by Default in Table S1) based on the maximum Q value; The ADMIXTURE assignment by default would be robust if the maximum Q value is significantly larger than 0.5, but would become ambiguous or arbitrary when the maximum Q (for group A) is near or less than 0.5 and is equal or very similar to the second large Q value (for group B). To solve the problem, we developed and followed a guide or a set of rules below to assign an Adjusted Group ID to the inbred line: the inbred line would be assigned into Group A if Q_A_ > 0.5 and Q_A_-Q_B_ > 0.1, and it would be assigned into Group A_Para if Q_A_ > 0.5 and Q_A_-Q_B_ < 0.1 or Q_A_ ≤ 0.5 and Q_A _− Q_B_ ≥ 0.15, otherwise, it would be assigned into Group Mixed. Here A and B is the group with the largest and second large Q value respectively (see the column: Group ID Adjusted in Table S1).

## Results

### SNP characteristics

The number of SNP loci (SNPs), SNP density, and four genetic diversity parameters: MAF, GD, PIC, and H (Heterozygosity)) are plotted by chromosome for the un-filtered data (876,305 SNPs) and filtered data (525,141SNPs) in Fig. S1a and S1b respectively. For both datasets, Chr.1 has the highest number of SNPs and Chr. 10 has the least (133,871, and 61,924 for the un-filtered data in Fig. S1a, and 80,958 and 37,329 for the filtered data in Fig. S1b), whereas the difference in SNP density are less dramatic across different chromosomes, with Chr. 5 having the highest density and Chr. 4 having the lowest (Fig. S1a, 1b). The degree of genetic diversity measured by MAF, Heterozygosity, GD, and PIC are almost constant across chromosomes with mean value of 0.14, 1.5%, 0.2, and 0.17 for the un-filtered data (Fig. S1a) and 0.22, 2.01%, 0.31 and 0.25 for the filtered data (Fig. S1b), indicating that data filtering by TASSEL V5.2 does improve the data quality.

**Figure 1:**
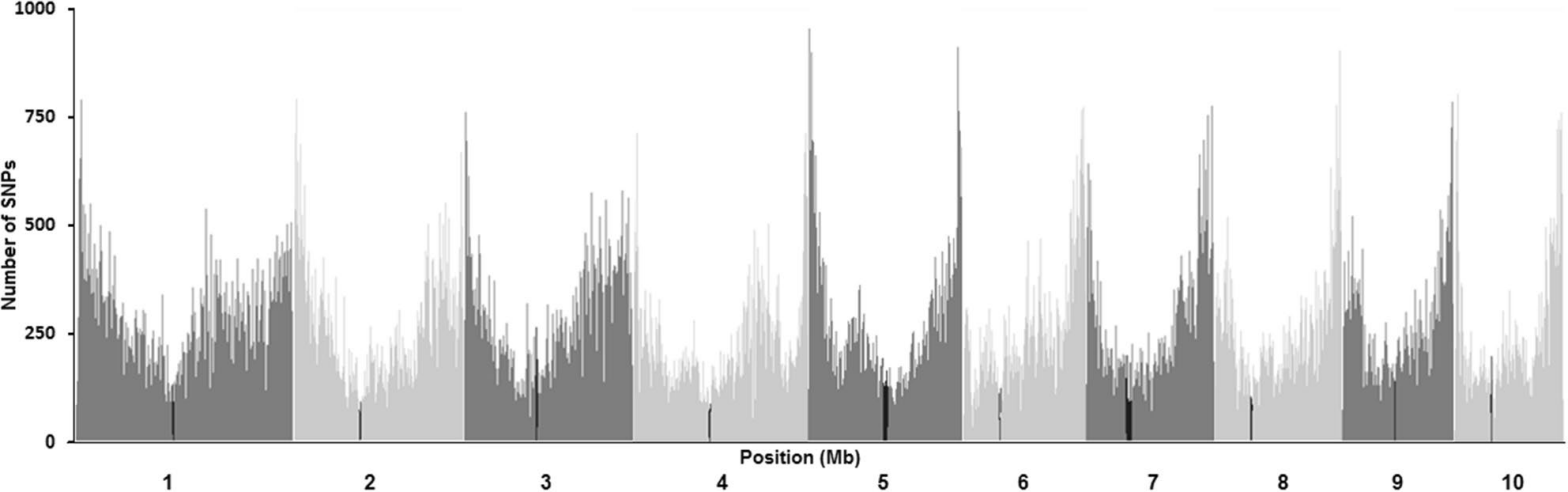
SNP density and distribution pattern on 10 chromosomes of maize genome.

**Figure 2:**
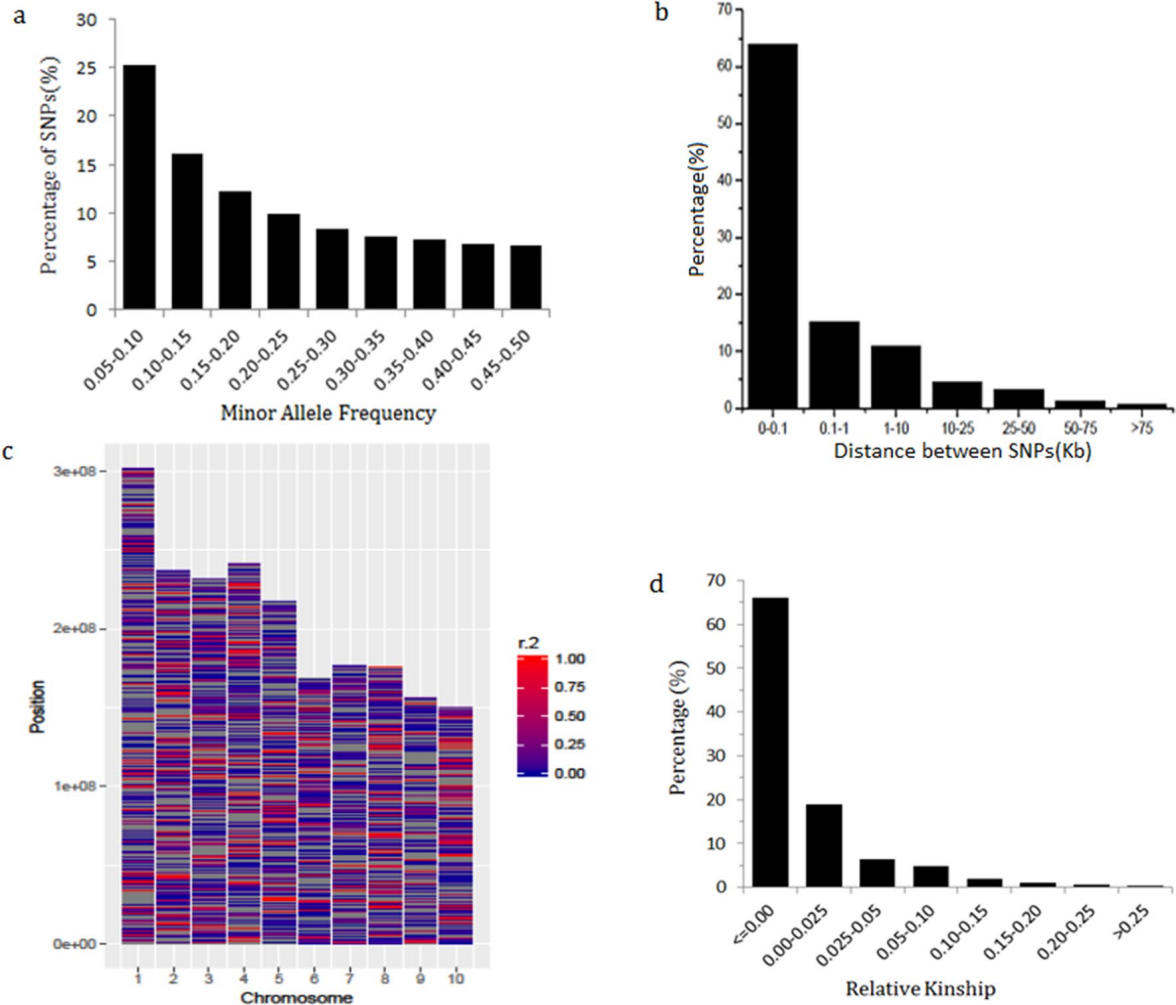
The molecular characteristics of maize genomes. (**a**) Minor allele frequency distribution; (**b**) Physical distance between adjacent SNP loci; (**c**) LD pattern of SNP loci along each of the 10 chromosomes; (**d**) The Relative kinship of 490 inbreds.

For the data of 525,141 SNP loci, the pattern of SNP density along each chromosome is similar for all 10 chromosomes: low near centromere region and high near telomere region (Fig. 1), Chromosome 1, 4, 5, and 7 also show a small third peak between centromere and telomere. The minor allele frequency (MAF) (Fig. 2a) shows a skew distribution with 41% of loci having MAF between 0.05 and 0.15 and only 20.6% between 0.35 and 0.50. The physical distance between two adjacent SNP loci on a chromosome measured by Kb also has a skew distribution (Fig. 2b), with about 64% of SNP loci having distance between 0 and 0.1 kb and about 15% of SNP loci between 0.1- 1.0 kb, and only about 0.06% of SNP loci having distance larger than 75 Kb, indicating that about 94% of maize genomes of 490 inbred lines are well-covered by 525,141 SNP loci.

### Linkage disequilibrium

Linkage disequilibrium (LD) analysis was carried out for 525,141 SNP loci using TASSEL5.2 software. The segment average LD vary along a chromosome, with high and low LD segments or regions alternate as red (high LD) and blue (low LD) colored bands or stripes of different length (Fig. 2c). The average LD measured by r^2^ for each chromosome is between 0.123 and 0.137 (Table 1). The average LD decay distance, defined by the increase of physical distance between adjacent SNP loci when LD decrease from r^2^ = 1.0 to r^2^ = 0.1, ranges from 35 kb (Chr. 8) to 245 kb (Chr. 9). Chr. 8 and Chr. 2 have the shortest LD decay distance (35 kb, 55 kb), smallest mean value of ${\mathrm{r}}^{2}$ (0.123, 0.125) and highest rate of LD decay (Table 1, Fig. S2), Chr. 9 has the longest LD decay distance (245 kb), the second large mean ${\mathrm{r}}^{2}$(0.133) and lowest rate of LD decay (Table 1, Fig. S2). Overall, LD decays very fast when ${\mathrm{r}}^{2}$ is between 0.22 and 0.11 and slow down and become flat when ${\mathrm{r}}^{2}$ is close to 0.1 (Fig. S2).

### Relative kinship

TASSEL5.2 was used to compute Relative kinship. Majority of the pairwise relative kinships (66% ) are equal or close to 0, only 9% of them are above 0.05 (Fig. 2d), an indication that most inbred lines in the collection of 490 inbred lines from CSM region are either not related or only distantly related to each other, therefore the maize germplasm at CSM region is genetically very diverse.

### Group and subgroup identification and validation

ADMIXTURE 1.3.0 software package^31^ was used to detect population structure and to assign 490 inbreds into K groups using 4849 tagSNPs (see Table 1).

**Figure 3:**
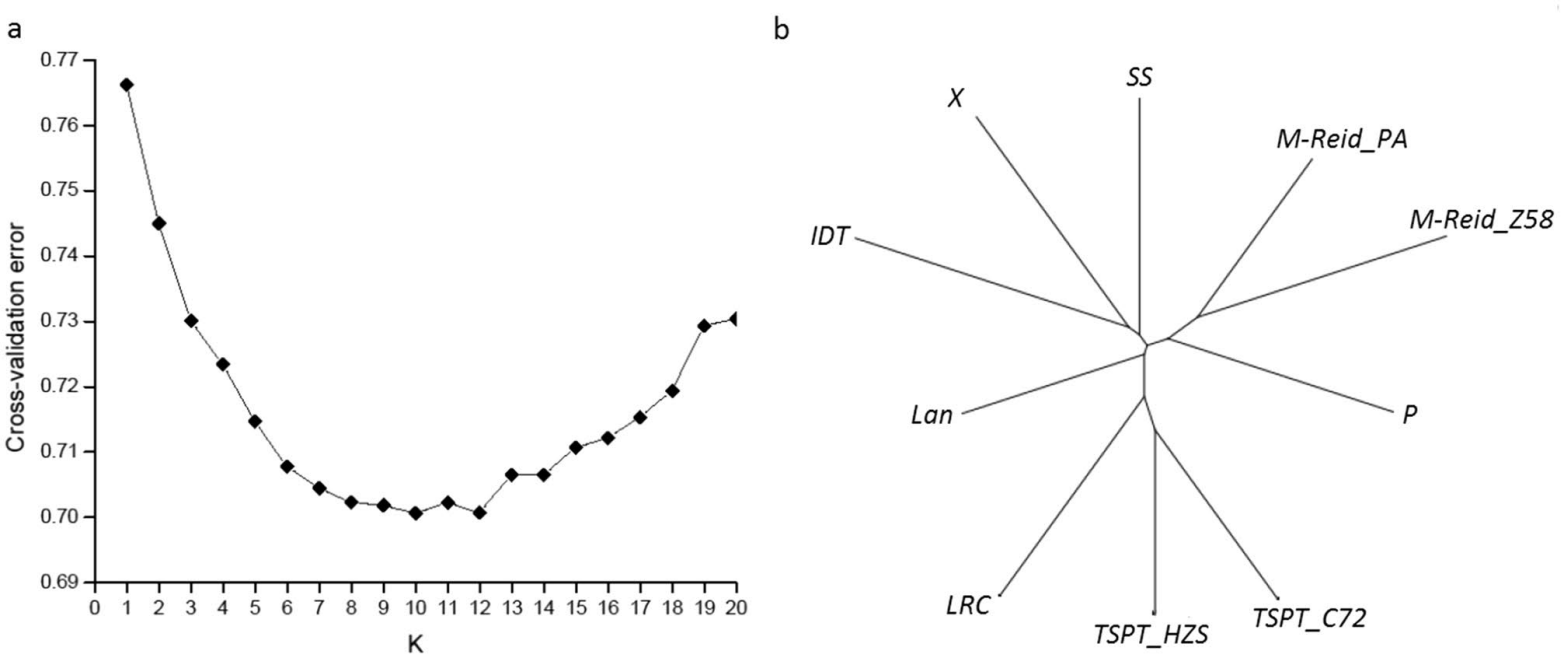
The cross-validation error with different K. (**a**) the best K value is 10; (**b**) Genetic differentiation among 10 subgroups based on pair-wise Fst estimates.

To identify the optimal K, K values ranging from 1 to 20 were set up to run ADMIXTURE 1.3.0 software, the cross-validation error curve based on ADMIXTURE output (Fig. 3a) , shows that cross-validation error value of 0.701 was smallest at K = 10 (Fig. 3a), thus, dividing the 490 inbred lines into 10 groups (that is, 10 heterotic subgroups) is optimal.

Table S1 is the ADMIXTURE output at K = 10. Each inbred line was assigned a set of 10 Q values and a Group ID or heterotic subgroup ID by Default based on the maximum Q value (Table S1). An adjusted Group ID (called Group ID Adjusted in Table S1) was also assigned to the inbred line if its maximum Q value is near or less than 0.5, indicating that the group identity of that inbred is ambiguous (see Material and Methods for the guide of assigning Adjusted Group ID).

To validate the group identity or group membership assigned by ADMIXTURE 1.3.0, the following two approaches were taken: (1) PCA plot visualization at 2-D space and (2) comparing the group identity or affiliation assigned by ADMIXTURE with that based on other independent prior knowledges for a set of well-known core or indicator inbreds.

**Figure 4:**
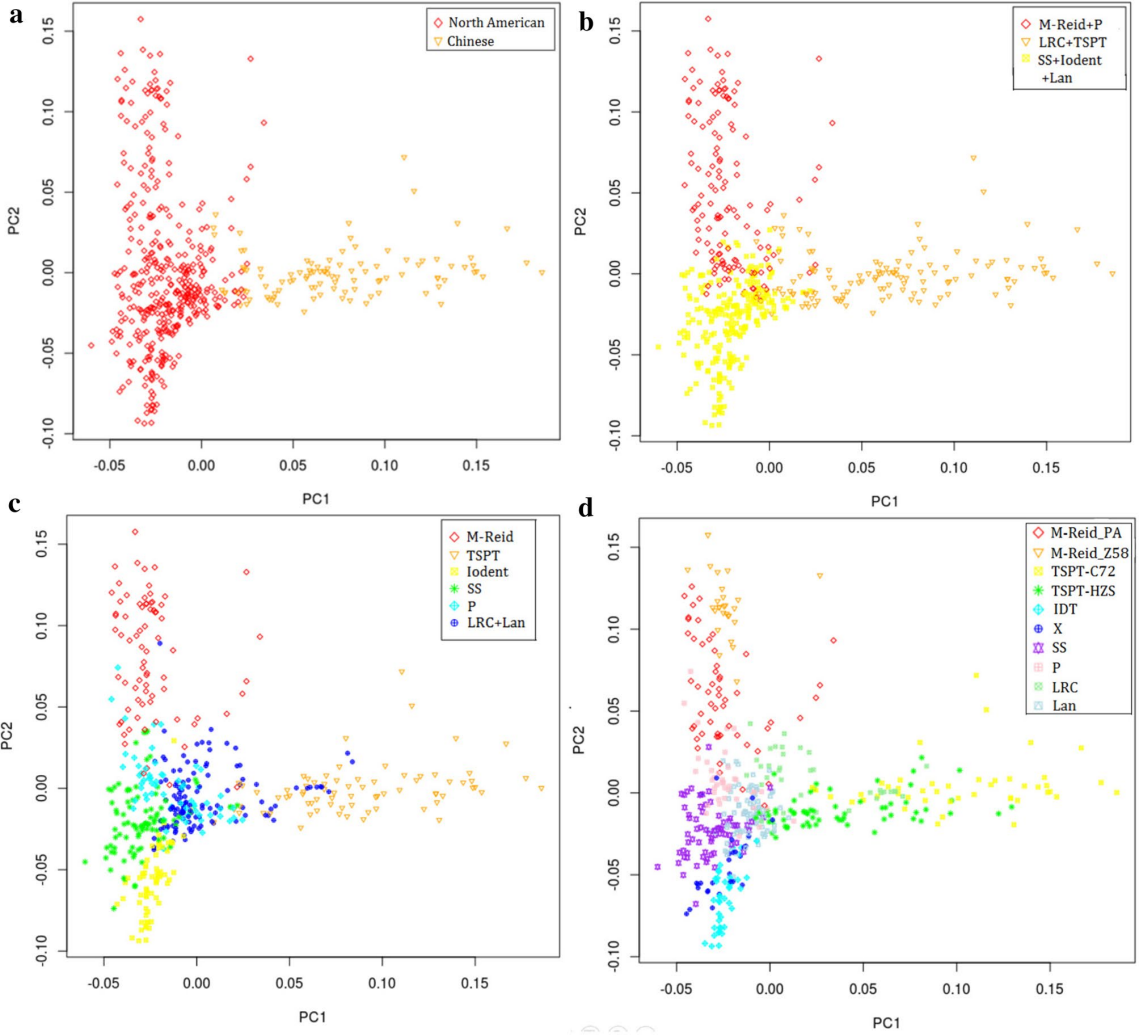
The 2-D PCA Plots of the ADMIXTURE output for 490 maize inbred lines generated by SNPRelate. (**a**) K = 2; (**b**) K = 3; (**c**) k = 6; (**d**) k = 10.

The ADMIXTURE output was visualized on PCA plots at K = 2, 3, 6, and 10 (Fig. 4). The clear boundary among different groups and very few overlapping and outliers indicate that ADMIXTURE has done a good job in group delineation. As Fig. 4 shows, at K = 2, the 490 inbreds were grouped into two groups, named Chinese group and North American group (Fig. 4a); at K = 3, the Chinese group (LRC + TSPT) remained unchanged and the North America group was further delineated into two groups: M-Reid + P and SS + Iodent + Lan. The three groups appear as a triangle or a delta at 2-D space (Fig. 4b); at K = 6, the TSPT, M-Reid, and Iodent appear at the tips of the triangle and P, SS, and LRC + Lan are located near its center (Fig. 4c). From K = 6 to K = 10, LRC and Lan become separated groups and the three groups, M-Reid, TSPT, and Iodent, split further into two for each, to form three subgroup pairs or 6 subgroups: M-Reid_PA and M-Reid_Z58, TSPT_C72 and TSPT_HZS, and IDT and X (Fig. 4d). The subgroup splitting pattern is corroborated by the branching pattern of Fst tree on Fig. 3b, where M-Reid, TSPT, and Iodent all were bifurcated further into two terminal branches each.

The ADMIXTURE output was further validated by comparing the group affiliation assigned by ADMIXTURE and that established by other independent prior knowledge for a set of inbred lines, which we call the core inbreds or indicator inbreds of a heterotic group or subgroup. These set of inbreds are called indicator inbreds because their heterotic group affiliation are known and were established based on independent prior knowledge, including genetic pedigree information , results of field combing ability tests, and the consensus of a majority of corn breeders.

**Figure 5:**
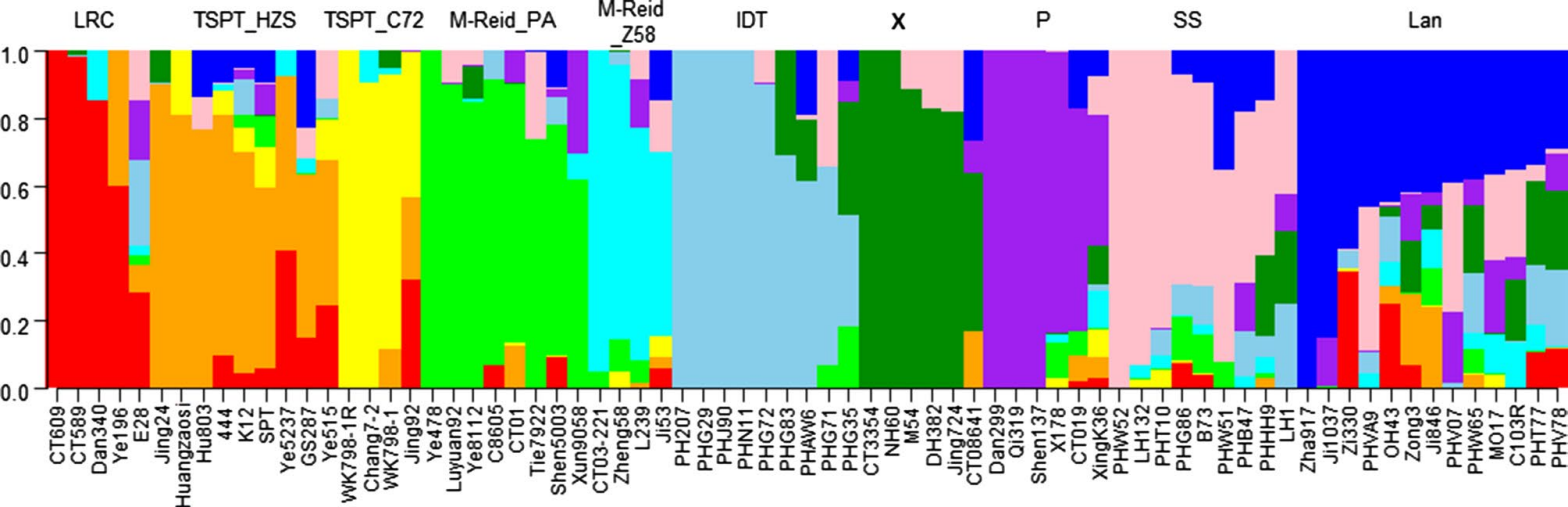
The subgroup assignment by ADMIXTURE for a set of indicator inbred lines when K = 10.

The results show that ADMIXTURE did assign these inbreds correct group affiliations (Fig. 5). Here only a subset of the indicator inbreds for each of the 10 heterotic subgroups and their maximum Q value from ADMIXTURE are listed: LRC (CT609, 1.0; Dan340, 0.85), TSPT_HZS (444, 0.71; TSPT, 0.54 ), TSPT_C72 (Chang7-2, 1.0; Xun92-7, 0.62), M-Reid_PA (Ye478, 1.0; Tie7922, 0.73; Shen5003, 0.68), M-Reid_Z58 (Zheng58, 0.81; Ji53, 0.54), X (CT3354, 1.0; DH382, 0.83; Jing724, 0.82), IDT (PH207, 1.0; PHG72, 0.90), P (Qi319, 1.0; X178, 0.83), SS (PHW52, 1.0; LH132, 0.93; B73, 0.6), and Lancaster (Ji1037, 0.85; Zi330, 0.59).

Table S1 gives the group affiliation (Group ID, Adjusted Group ID) and Q values of all 490 inbred lines at K = 10 and K = 6. The Group ID by default was assigned by ADMIXTURE for each inbred based on the maximum Q value (Table S1); for some inbreds, an Adjusted Group ID for either a para group or a mixed group would also be assigned if the Group ID by default is not reliable (see Material and Methods for the guide of Adjusted Group ID assignment). For example, inbred PHJ33 (IDT, 0.35; X, 0.26) and PHG30 (IDT, 0.33; X, 0.33; M-Reid_PA, 0.18) were both assigned into IDT subgroup at K = 10 by default but were assigned into IDT_Para subgroup and Mixed subgroup respectively based on our group assignment guide.

### Genetic and breeding features of different heterotic groups

**Figure 6:**
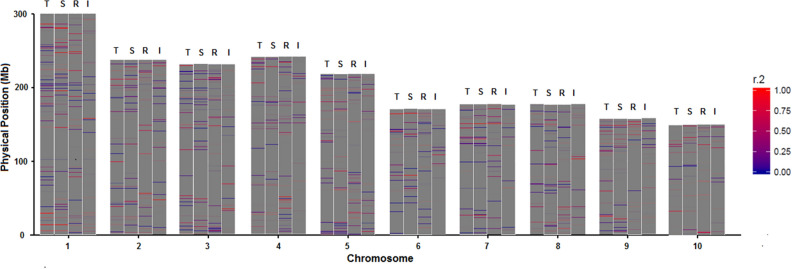
LD block pattern of four subgroups. TSPT (T), SS (S), M-Reid (R), and Iodent (I).

PCA plots at Fig. 4 and the Fst tree on Fig. 3b clearly illustrate the pathway of reverse coalescence or branching out from 2 mega groups, then 3 supergroups, 6 groups, and ending at 10 subgroups. The proportion and the number of inbreds included in each group and subgroup are shown at Fig. 8a, 8b and Table S3, group-specific genome diversity (Table S3, S4) and group-specific LD block pattern are shown at Fig. 6. Some genetic and breeding features of them are summarized below.

#### TSPT group

Its two subgroups TSPT_C72 and TSPT_HZS takes up 9% and 13% respectively (Fig. 8b), they are descendents with various degree of affiliation from a well-known Chinese local germplasm line TangSiPingTou (TSPT), the division into two subgroup occurs during 2000s when hybrid Zhengdan958 becomes a big commercial hybrid at CSM region and many inbreds in TSPT_C72 subgroup are derived from its male parent Chang7-2 and are used as male parents of other big commercial hybrids in CSM region, such as, Xun 92–8 of hybrid Xuandan20, Chang7-2 of hybrid Zhongke 11, and Jing92 of hybrid Jingke968. Many inbreds in the TSPT_HZS subgroup serve as male parents of a number of big commercial corn hybrids of 1980s and 1990s, such as, HZS of hybrids Yedan 2 and Ye515 of hybrid YeDan 12 (Table S1). The genetic diversity within both TSPT_C72 subgroup (containing 46 inbreds) and TSPT_HZS (contain 63 inbreds) are large (GD are 0.301 and 0.333 respectively, Table S3).

#### Lancaster group

87 inbreds (18%, Fig. 8b) were grouped into this group by default, but 59 inbreds are reassigned into Mixed or Lan_para based on Adjusted Q score (Table S1), indicating that the heterotic group identity of many inbreds in this group is ambiguous and PCA plots also show that most inbreds in this group are located in the center of the delta, that is, in the intersection of different groups (Fig. 4c, d). The inbreds in this group have not significant parental contribution to big commercial hybrids in CSM region. 23 of total 74 inbreds from the Ex-PVP collection, including some well-known inbreds such as LH123 and LH51 are also grouped into Lancaster group. The genetic diversity within this group is large (GD = 0.332).

#### IDT and X subgroup

Among 41 inbreds in IDT subgroup, 29 are Ex-PVP inbreds, but none of the inbreds in X subgroup is directly originated from Ex-PVP. The IDT subgroup does not has significant presence in the germplasm until 2000s^8^ whereas the X group is a totally new group in CSM and in Chinese germplasm arising in 2000s^4,7–9,19^. The Ex-PVP inbreds in the IDT subgroup have very little direct use as parents in commercial hybrid creation, likely due to their poor adaptation to the local farm environment (e.g. severe diseases). Many inbreds in the X subgroup are female and male parents of big commercial hybrids at CSM region, such as, Jing724, M03, and NH60 as female parent of Hybrid Jingke968, Liangyu99, and Nonghua101 respectively, and DH382 as male parent of Hybrid Denghai605. IDT and X subgroup have the closest genetic affiliation with each other but are far from all other 8 subgroups revealed at the PCA plot and Fst tree (Fig. 3b, 4).

#### M-Reid group

The founder germplasm of M-Reid group was from the Reid germplasm of US and was introduced into China at 1950s to improve local maize lines, thus, their derivatives are called Modified-Reid or M-Reid in short. At 1970s and 1980s, new germplasm from the North American, in particular, from Pioneer Hybrids, such as, 78599, P3147, and P3382 were introduced into China to improve the disease resistance of M-Reid group, many inbreds of big commercial hybrids in CSM regions in 1980s and 1990s, such as, Ye478, Tie7922, and Shen5003 were created and they form the subgroup M-Reid_PA^2,8,15^. Many inbreds in M-Reid_Z58 subgroup are derived from inbred Zheng58, which was developed at 1990s, and became the parental inbred of Zhengdan958, the largest commercial hybrid at CSM region in 2000s and still has quite large acreage nowadays. The M-Reid_Z58 subgroup has the largest genetic distance from the IDT subgroup (Fst = 0.346) (Table S2 and Fig. 3b).

#### The Ex-PVP inbreds

The total 74 Ex-PVP inbreds in the CSM collection are grouped into three subgroups: IDT (29), SS (22), and Lan (23) (see Table S1). The group identity of many inbreds such as PH207, PHP02, LH82, agrees well with that reported by Mikel and Dudley^6^ using pedigree information and by Beckett et al.^18^ using GBS molecular marke data, suggesting that GBS SNP data can produce reliable heterotic group assignment. 265 inbreds in the 490 inbred lines collected at CSM region (about 54%), including all inbreds in subgroup IDT, X, P, Lan, and SS, have major contribution from North American germplasm and 28% of them (74/265) are Ex-PVP inbreds (Fig. 8b).

### Genome differentiation among subgroups

For the groups or subgroups assigned by ADMIXTURE, the following respects were examined molecularly: (1) genome differentiation with Wright’s Fst (2) linkage disequilibrium pattern (3) SNP Loci Polymorphism measured by GD and PIC (4) Heterotic group-specific SNP allele polymorphism.

**Figure 7:**
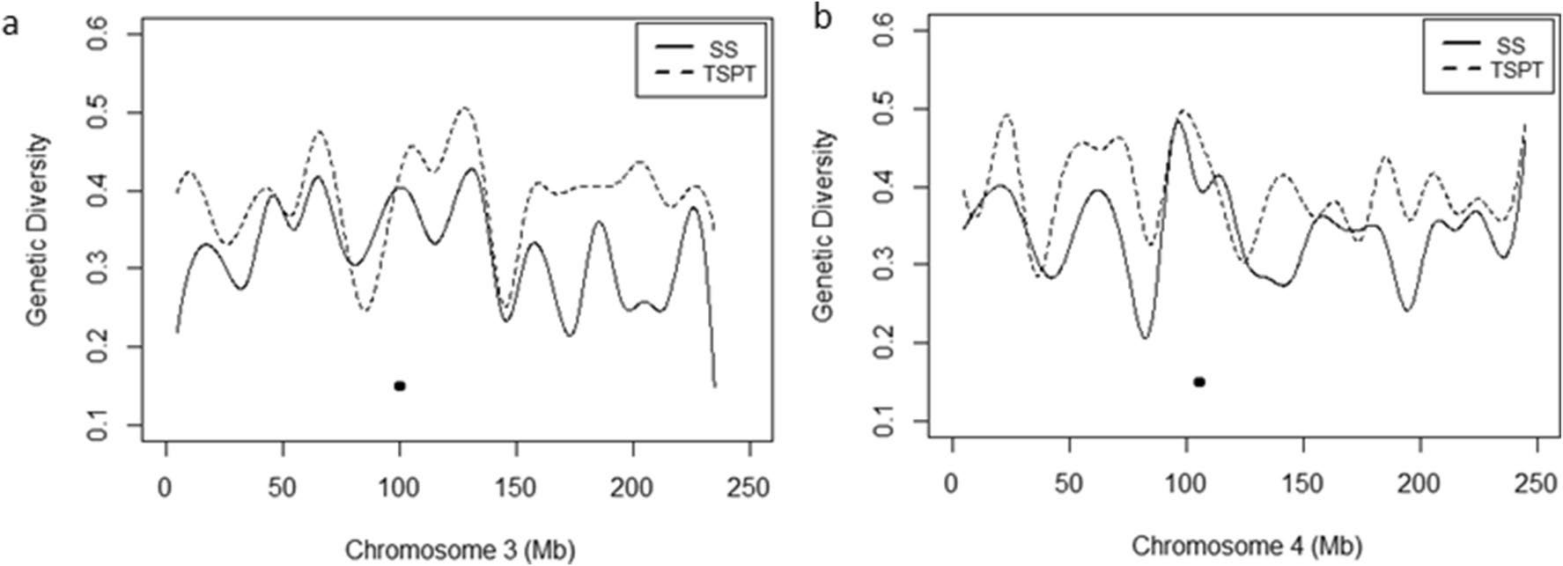
Genetic Diversity (GD) in SS and TSPT across chromosome 3 and chromosome 4.

1. *Wright’s F*_*st*_ The pairwise fixation indexes from Wright^29,30^, or Fst, was calculated using data from the 4849 tagSNPs loci summarized at Table S2 to measure the degree of genetic differentiation or genomic distance among 10 subgroups. As shown on Table S2 and Fig. 3b, M-Reid_Z58 subgroup and the IDT subgroup have the largest genomic differentiation or genomic distance (F_st_ = 0.346), and TSPT_C72 subgroup and the TSPT_HZS subgroup have the smallest genomic differentiation or genomic distance (F_st_ = 0.209). IDT has the largest mean F_st_ value (0.275) and Lan has the smallest mean Fst (0.228), indicating that IDT subgroup has the largest genomic distance from all other subgroups and Lan subgroup has the smallest genomic distance. These findings are consistent with the PCA results shown in Fig. 4 and with the branching pattern shown at Fig. 3b, where clearly the TSPT has the most distal bifurcation to form subgroup TSPT_C72 and TSPT_HZS.
2. *LD block distribution along chromosomes* Different subgroups have very different LD block distribution pattern (see Fig. 6). Similarity at some local regions of chromosomes do exist even between the two most distal subgroups: the Chinese germplasm subgroup TSPT (T) and North American germplasm subgroup SS (S), for both subgroups have high LD segments near 50 Mb physical position and low LD segments at 200 Mb physical position of Chromosome 1 (Fig. 6).
3. *SNP Loci Polymorphism* Within-subgroup SNP loci polymorphism measured by GD and PIC are reported in Table S3, TSPT subgroup has high level of polymorphism at K = 6 (with average = 1/2(GD + PIC) = 0.259), and at K = 10 (with average of 0.257 and 0.253 for subgroup TSPT_Z58 and TSPT_C72 respectively), the IDT and X have the smallest within-subgroup SNP loci polymorphism of 0.212 and 0.221 respectively at K = 10 (Table S3). The GD pattern of SNP loci polymorphism along chromosomes also differ for different subgroups, the chromosomal GD pattern for SS and TSPT subgroup are shown at Fig. 7. For Fig. 7, the average GD value of all SNP loci within a moving window of 10 Mb was plotted for each of the 10 chromosomes, high GD peaks and low GD valleys are observed in number of chromosome regions, the two subgroups have opposite pattern in some regions and similar pattern in other regions (see Chr. 3, Fig. 7a, Chr. 4, Fig. 7b).
4. *Heterotic group-specific SNP allele polymorphism* Heterotic group-specific SNP loci were compared among different groups, SNP loci that are unique, and that show neutral allele frequency in one group but show near fixed allele frequency in another group are common, only the comparison between M-Reid and TSPT are reported here (Tables 2, 3) because these two subgroups form the most important female and male heterotic group pair or heterotic pattern (M-Reid × TSPT) at CSM region; a large number of commercial corn hybrids in CSM regions were created following the M-Reid × TSPT pattern, including the dominant corn hybrid, ZhengDan 958 (M-Reid × TSPT), which took up 30% of acreage of corn production at CSM region at its peak years in 2000s.

**Table 2:** Number of unique, neutral or fixed SNP loci in M-Reid and TSPT group.

Chromosome	No. of unique SNP M-Reid	No. of unique SNP in TSPT	No. of neutral SNP in M-Reid and fixed SNP in TSPT	No. of neutral SNP in TSPT and fixed SNP in M-Reid
1	6	8	18	18
2	7	9	15	11
3	6	1	8	20
4	2	7	16	22
5	4	5	6	10
6	4	3	17	9
7	4	6	8	11
8	6	6	14	14
9	3	0	6	26
10	1	1	11	8
Total	43	46	119	149

**Table 3:** SNP loci with heterotic group-specific allele frequency difference (M-Reid and TSPT).

SNP name	Chr^a	Allele	Allele fequency (P)		P_M-Reid-TSPT_	SNP name	Chr^a	Allele	Allele fequency (P)		P_M-Reid-TSPT_
			M-Reid	TSPT					M-Reid	TSPT	
S1_45003478	1	C	0.945	0.213	0.732	S8_119990618	8	A	0.953	0.230	0.723
S2_54669791	2	C	0.250	0.756	− 0.506	S8_156241083	8	G	0.730	0.058	0.672
S2_176020217	2	A	0.798	0.063	0.735	S8_162588367	8	T	0.061	0.942	− 0.881
S2_191772106	2	T	0.934	0.263	0.671	S8_162785756	8	A	0.078	0.795	− 0.717
S2_198437096	2	T	0.061	0.779	− 0.718	S10_9987855	10	T	0.865	0.163	0.702
S2_226557508	2	C	0.122	0.770	− 0.648	S10_83778123	10	G	0.939	0.244	0.695
S2_226557527	2	G	0.894	0.094	0.800	S10_141271856	10	C	0.078	0.945	− 0.867
S3_214980179	3	A	0.775	0.049	0.725	S10_144619447	10	G	0.197	0.867	− 0.670
S4_161036243	4	G	0.068	0.838	− 0.770	S10_144979499	10	G	0.838	0.094	0.744
S6_18591062	6	T	0.182	0.867	− 0.685	S10_147013864	10	G	0.881	0.205	0.676

## Discussion

### Chromosomal density and linkage disequilibrium pattern of SNP loci

The SNP density along chromosomes is  much higher near telomere region than near centromere region, this pattern persists for all 10 chromosomes. Similar distribution patterns we observed (Fig. 1) were also reported by Romay et al.^14^ (Fig. 1) and Martins et al.^32^. The underlying genomic mechanism determining the observed SNP loci distribution pattern is unclear and it has been known that the distal regions of a chromosome turn to have relatively high level of genetic recombination and are less methylated than the pericentromeric regions (the region near centromere)^32,33^. LD decay very rapidly on all 10 chromosomes and reached r^2^ = 0.11 at about 20 Kb on average (Fig. S2), similar rate of LD decay was also reported by Romay et al.^14^. Variation in SNP density and LD are also observed at different chromosome segments or regions, at different chromosomes, and in different heterotic groups (Figs. 2c, 6).

Majority (64%, or 336,331) of SNP loci reside very closely physically on chromosome (distance interval between 0 and 0.1 kb, (see Fig. 2a) whereas only 22.41% (117,698) of SNP loci, have very high LD (r^2^ ≥ 0.8, Fig. 2b), this discrepancy between physical distance and LD indicates that many SNP loci that reside very closely to one another physically do not have high LD, therefore they are likely located at the chromosome segments called hot spots of genetic recombination. The presence of hot spots of genetic recombination is also indicated by the presence of red (high LD) and blue (low LD) bands or stripes along each chromosome we observed (Fig. 2c) and similar observations were also reported by Thirunavukkaraasu et al.^34^ through linkage disequilibrium heatmap and by Martins et al.^32^ through FISH chromosome painting.

### SNP loci polymorphism

In the 876,305 SNP loci we identified from 490 inbred lines, 40% are rare allele loci (MAF < 0.05). Ramoy et al.^14^ reported that over 50% of SNP loci have rare alleles in the USDA collection of 2711 inbred accessions. The average minor allele frequency for the filtered data set (525,141 SNP loci) is MAF = 0.22, Wu et al.^35^ reported an average MAF = 0.22 for the 362,008 SNP loci data set collected using GBS sequencing of 538 CIMMYT maize inbred lines. Since SNP loci is bi-allelic, the above result indicate that the frequency of two alleles of a SNP locus is p = 0.22 and q = 0.78 respectively on average, far from the allele frequency p = q = 0.5 expected from a randomly mating Mendelian population with two neutral alleles; the high percentage of rare allele loci and the lower average MAF (far from MAF = 0.5) are all strong indications of natural and artificial selection that drive the formation of population structure in the CSM germplasm. Further analysis of allele frequency difference between subgroups show the existence of the subgroup-specific SNP loci, these loci are neutral or have frequency near 0.5 in one subgroup but were fixed or nearly fixed (allele frequency near to 0) in another group (Tables 2, 3), the above observations all indicate that different heterotic groups are under different selection forces, likely applied by researchers with different breeding goals for different heterotic group, in particular, for male and female parental heterotic groups^1,10,36,37^. It should be pointed out that here we are using all the terms from population genetics and evolutionary biology, such as neutral allele, fixation, selection, and various diversity measurements just for statistical convenience, since a collection of diversity panel of maize inbreds is not a typical randomly mating maize population^11,38^.

The Genetic Diversity, or GD, measures difference in frequency between two alleles in SNP loci. The entire inbred panel of 490 inbreds has average GD = 0.344 and the 10 subgroups have GD ranging from 0.277 to 0.333 (Table S3). The GD values also change along chromosome and different subgroups could have different chromosomal pattern (Fig. 7). Similar GD values were also reported by Wu et al.^15^ and Zhao et al.^19^.

### Grouping maize germplasm using haplotype and TagSNPs

To reduce the cost of genome DNA sequencing in SNP loci discovery and the cost of genotyping in applications to germplasm study and molecular breeding, several molecular marker technologies have been developed, including but not limited to: GBS, GBTS, SLAF-seq, SNP Chip, et al.^22,28,39^. In this study, GBS technology developed by Elshire et al.^22^ was adopted to produce high quality SNP data, and Haploview v4.2 was used to identify 4849 tagSNPs sites from 525,141 SNP markers loci of GBS data, the combination of these two technologies make development of a set of high quality SNP markers without significant loss of information possible. Wu et al.^15^ shows that 700–1000 SNPs were necessary to robustly estimate the genetic difference among subpopulations, our result shows that about 5000 tagSNPs and haplotype loci derived from them are suitable for population structure analysis and germplasm study. In maize and many other organisms, it has also been reported that haplotype loci derived from tagSNPs are more informative than binary SNPs^16,40,41^.

### Population structure and subgroup differentiation

The ADMIXTURE output and PCA representation all show clearly the existence of population structure in the CSM collection and the 490 inbreds can be grouped into 3 supergroups, 6 groups and 10 subgroups with clear boundary and very little mixing. Fixation Index (Fst) was calculated to measure the subgroup differentiation and the F_st_ value between any two subgroups ranges from 0.209 to 0.346. According to Wright’s guide^29^, two populations with F_st_ > 0.25 have very large genetic differentiation and with 0.15 < Fst < 0.25 have large genetic differentiation. Therefore, our F_st_ results indicate that there is very large genomic differentiation between different heterotic groups at the CSM collection. Zhao et al.^19^ find the heterotic groups in Northern China have F_st_ value: 0.325–0.457 using SNP chip data and 344 inbreds.

### Heterotic group assignment using genomic data

Traditionally, breeding pedigree information is used to determine the parental contribution and predict the heterotic group affiliation of a particular inbred line, but it is not possible to know the exact proportion of genome contribution from a parent or a progenitor to the inbred line solely based on pedigree information because trait selection applied by breeders at every step of the breeding process could change the proportion. Here we show that genome data can do a better job. For example, Inbred LH132 and LH1 are taken as SS germplasm because they both are derived from SS core inbred B73^42^. Based on pedigree information, B73 has contributed 3/4 or 75% of genome to both LH132 [(H93xB73)/B73] and LH1[(B73xH644)/B73] , but based on genomic DNA sequence data (ADMIXTURE output, Table S1), LH132 has 93% SS affiliation (SS, 0.93) and LH1 has only 43% SS affiliation (SS, 0.43; IDT, 0.25; X, 0.22; P, 0.11). Similarly, based on pedigree information, PHG83 (PH814xPH207) and PHG71(A632HtxPH207) are labeled as (IO/LAN/UR) and IO/SS respectively by Mikel^42^, but exact proportion of IDT (IO) contribution is unknown, from ADMIXTURE output, the IDT contribution to PHG83 (IDT, 0.69; X, 0.31) and PHG71 (IDT, 0.58; SS ,0.35) are 0.69 and 0.58 respectively. For many inbred lines, pedigree data are either missing or misreported, genomic data can provide important information on their heterotic group affiliation.

### Germplasm basis and heterosis utilization trend at CSM region

**Figure 8:**
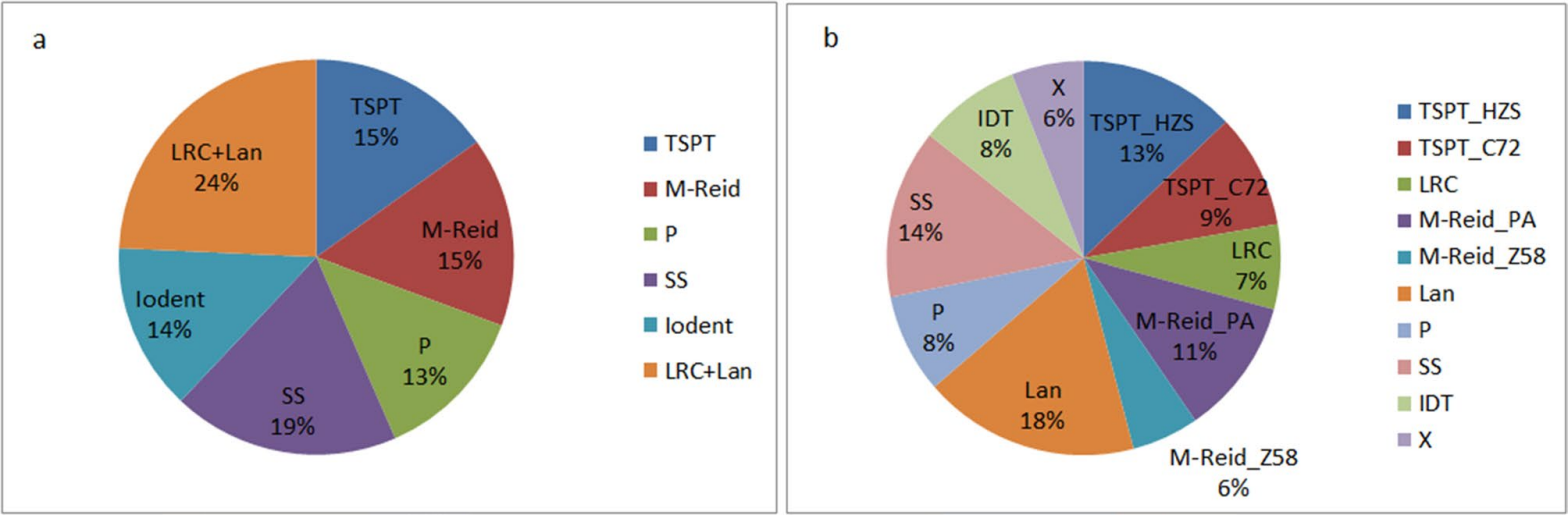
Heterotic groups of 490 inbred lines in CSM region. (**a**) K = 6; (**b**) K = 10.

In this study, Relative kinship analysis (Fig. 2d) shows that the maize germplasm at CSM region is genetically very diverse and more than 66% of 490 inbred lines have near zero kinship. Evidence from ADMIXTURE population structure analysis, PCA visualization, pedigree information validation, and population differentiation F_st_ analysis (Fig. 3b) suggest that the 490 inbreds can be grouped into 3 supergroups (LRC + TSPT, M-Reid + P, and SS + Iodent + Lan), 6 groups (Fig. 8a), and 10 subgroups (Fig. 8b). The three supergroups, as illustrated by PCA plots, appear as a triangle in 2-D space (Fig. 4b). The LRC + TSPT supergroup is named by two well-known founder lines or landraces of modern Chinese germplasm, LRC and TSPT. The M-Reid + P supergroup: includes M-Reid group (see Result) and many inbreds from the P group, which was developed at 1970s and 1980s by introgressing disease resistance genes from the North American germplasm, in particular, those from Pioneer Hybrids, such as, 78599, P3147, and P3382 into Chinese germplasm. The SS + Iodent + Lan supergroup contains many inbreds developed by US public research organizations, such as, B73 (SS) , Mo17 (Lan) in 70s and 80s and many ex-PVP inbreds introduced after 2000s, such as PH207 (Iodent)^14,19,43^.

The Predominant heterotic pattern in the last two decades in CSM region is Introduced × Local, which is also true in Chinese spring maize regions^2,19^. There are two concrete types of Introduced × Local: M-Reid subgroup × TSPT subgroup (Type I) , and X subgroup × Local subgroups (Type II). The Type I is exemplified by Zhengdan 958 [Zheng 58 (M-Reid) × Chang 7–2 (TSPT)] and a number of other hybrids with large commercial utilization: XunDan 20 (Xun9058 × Xun92-8), YeDan 2 (Ye107 × HZS), YeDan 12 (Ye478 × Ye515), and Zhongke 11 (CT03 × Chang7-2)^44^. The dominant presence of hybrid Zhengdan 958 at CSM commercial corn production (took up to 30% of corn planting area at its peak years of 2000s) leads to the increasing use of M-Reid_Z58 and TSPT_C72 breeding germplasm which are mostly derived from two inbred parents of Zhengdan 958: Zheng58 (Z58 for short) and Chang 7-2 (C72 for short) in inbred breeding programs in CSM region. Subgroups M-Reid_Z58 and TSPT_C72 count about 6% and 9% respectively in CSM breeding germplasm. The Type II, that is, X subgroup × Local subgroups, is exemplified by corn hybrid DH605 (DH351 × DH382, Nonghua 101 (NH60 × S121), Jingke 968 (Jing724 × Jing 92), and Liangyu 99 (Mo3 × M5972), many of them also have large presence in China Spring maize region.

In the past two decades, corn hybrid grain yield per unit area has increased more than two folds in CSM region and corn germplasm basis and heterotic pattern at CSM region has changed significantly and North American germplasm become more important at CSM region. Wang et al.^7–9^ have studied the CSM germplasm during later 1980s and early 1990s and has identified four major heterotic groups at CSM region and their relative proportion: M-Reid (25.6%), Lancaster (25.6%), TSPT (16.2%), LRC (10.7%), counting about 78% of the CSM germplasm^7–9^, whereas the four groups M-Reid (15.1%), TSPT (15.1%), LRC + Lan (24%), count about 54% of total germplasm now (Fig. 8a), the 24% drop during the past two decages is due to the significant increase of four heterotic subgroups of North American origin: P(8%), SS (14%), IDT (8%), and X (6%) (Fig. 8b), counting about 32% of total germplasm in CSM region.

## Supplementary information

Supplementary information 1.

Supplementary information 2.

Supplementary information 3.
