# Supplementary material for: Genetic variation and population structure in China summer maize germplasm
Source: Sci Rep. 2021 Apr 13;11:8012. doi: 10.1038/s41598-021-84732-6 (PMC8044188; doi:10.1038/s41598-021-84732-6)
Supplement: Supplementary file 1 — Supplementary information 1. [file 41598_2021_84732_MOESM1_ESM.docx]

**Supplementary Figure S1-S4**

**Genetic variation and population structure in China summer maize germplasm**

Guoping Shu^1✉,4^Email: [xugp2011@163.com](mailto:xugp2011@163.com),

Gangqiang Cao, Email caogq@zzu.edu.cn^2,4^

Niannian Li^2,4^, Aifang Wang^1^, Fang Wei^2,4^, Ting Li^1^, Li Yi^1^, Yunbi Xu^3^

Yibo Wang,Email: chigohut@163.com^1,4,5^

^1^Center of Biotechnology Beijing Lantron Seed, Zhengzhou,450001 Henan,China

^2^School of Agricultural Science Zhengzhou University, Zhengzhou, 450001 Henan, China

^3^Institute of Crop Science National Key Facility of Crop Gene Resources and Genetic Improvement, Chinese Academy of Agricultural Science, Beijing, 100081 China

^4^Zhengzhou University Graduate Student Training Base at Beijing Lantron Seed, Zhengzhou, 450001 China

^5^Henan LongPing-Lantron AgriScience & Technology Co., LTD, Zhengzhou, 450001 Henan, China

**
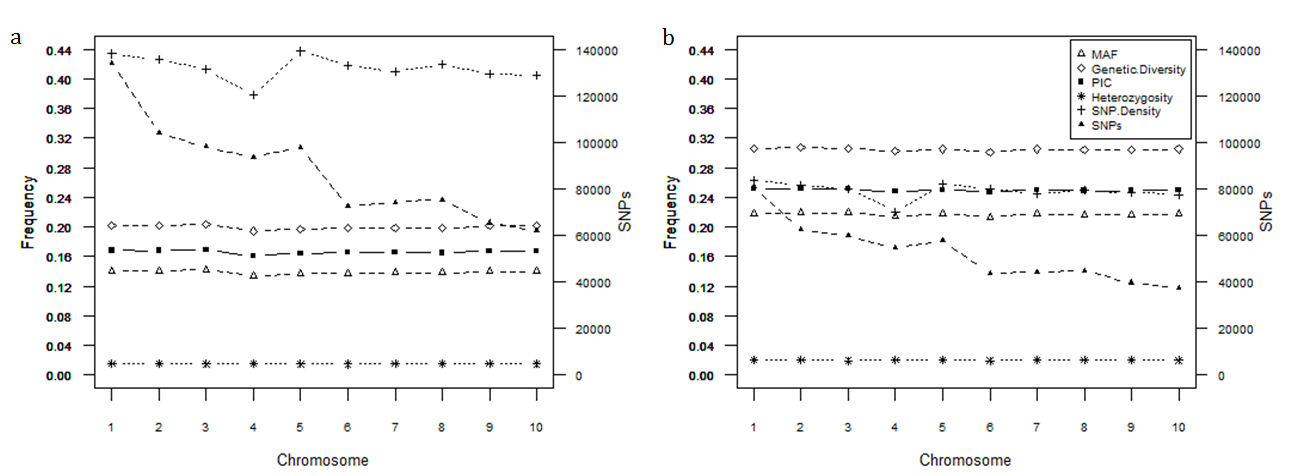
**

**Fig. S1** Summary statistics of SNP data collected from 490 inbred lines. a. The data set of 876,305 SNP loci; b. the data set of 525,141 SNP loci.


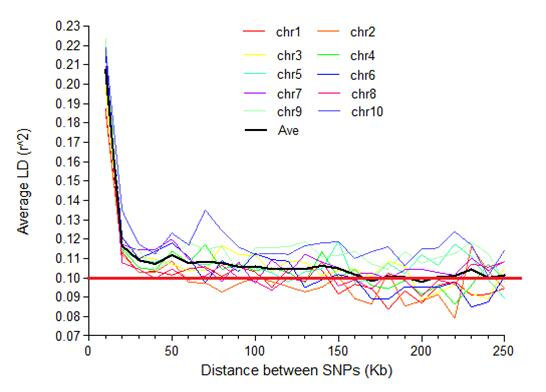


**Fig. S2** LD Decay with Distance between adjacent SNP loci.

**
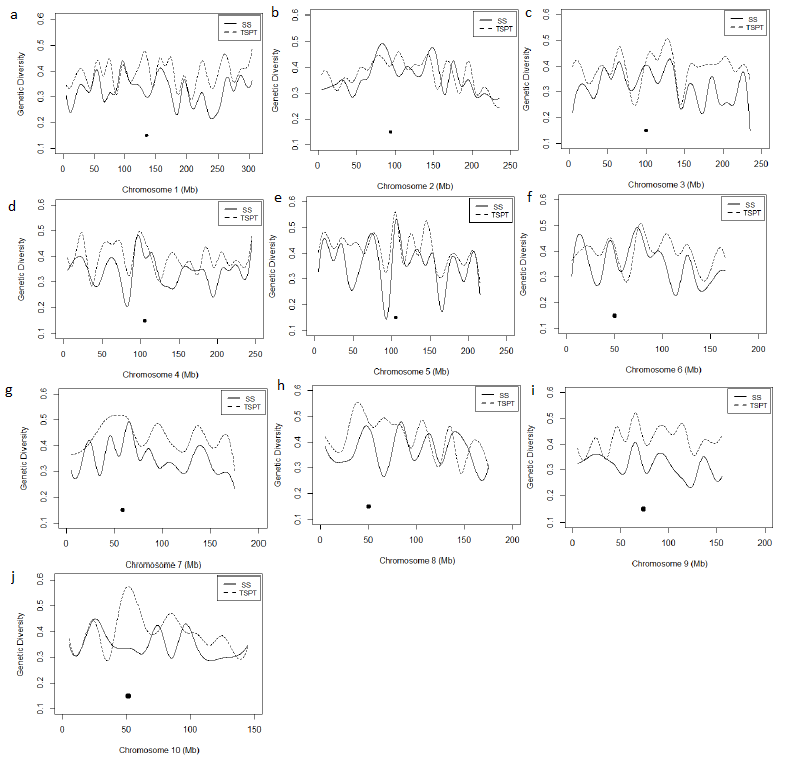
**

**Fig. S3** GD distribution of whole genome with 10-Mb bin size in SS and TSPT subgroups, black spots show the location of centromere. a-j show Chromosome 1-10,respectively.

**
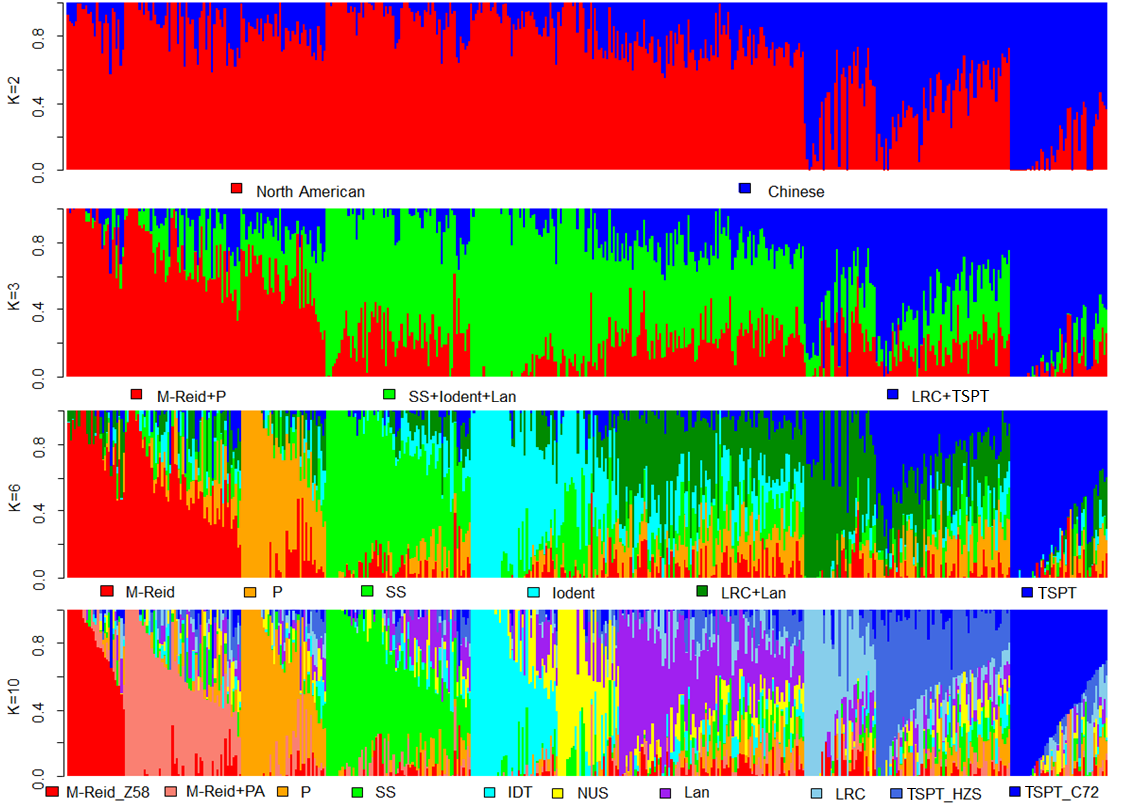
**

**Fig. S4** The clustering output of Admixture with K= 2, 3, 6 and 10
