# Supplementary material for: Genetic variation and population structure in China summer maize germplasm
Source: Sci Rep. 2021 Apr 13;11:8012. doi: 10.1038/s41598-021-84732-6 (PMC8044188; doi:10.1038/s41598-021-84732-6)
Supplement: Supplementary file 3 — Supplementary information 3. [file 41598_2021_84732_MOESM3_ESM.docx]

| Table S2 Pairwise Fst values between the ten subgroups | | | | | | | | | | |
| --- | --- | --- | --- | --- | --- | --- | --- | --- | --- | --- |
|  |  |  |  |  |  |  |  |  |  |  |
| Subgroup | M-Reid_PA | M-Reid_Z58 | TSPT_C72 | TSPT_HZS | IDT | X | SS | P | LRC | Lan |
| M-Reid_PA | 0 |  |  |  |  |  |  |  |  |  |
| M-Reid_Z58 | 0.242 | 0 |  |  |  |  |  |  |  |  |
| TSPT_C72 | 0.298 | 0.319 | 0 |  |  |  |  |  |  |  |
| TSPT_HZS | 0.273 | 0.317 | 0.209 | 0 |  |  |  |  |  |  |
| IDT | 0.309 | 0.346 | 0.32 | 0.308 | 0 |  |  |  |  |  |
| X | 0.284 | 0.323 | 0.322 | 0.301 | 0.29 | 0 |  |  |  |  |
| SS | 0.249 | 0.306 | 0.294 | 0.281 | 0.294 | 0.261 | 0 |  |  |  |
| P | 0.238 | 0.297 | 0.282 | 0.274 | 0.305 | 0.283 | 0.274 | 0 |  |  |
| LRC | 0.303 | 0.3 | 0.261 | 0.248 | 0.318 | 0.322 | 0.288 | 0.3 | 0 |  |
| Lan | 0.248 | 0.272 | 0.253 | 0.239 | 0.261 | 0.258 | 0.239 | 0.248 | 0.257 | 0 |
| Average | 0.244 | 0.272 | 0.256 | 0.245 | 0.275 | 0.264 | 0.249 | 0.250 | 0.260 | 0.228 |

| Table S3 Genome Diversity of Entire Panel and Each Subgroup | | | | | | |
| --- | --- | --- | --- | --- | --- | --- |
|  |  |  |  |  |  |  |
| Grouping | Subgroup Name | No.of lines | Genetic Diversity （GD） | Heterozygosity Rate (HR) | PIC | Average |
|  | Entire panel | 490 | 0.344 | 0.178 | 0.277 | 0.266 |
| K=6 | M-Reid | 76 | 0.321 | 0.196 | 0.259 | 0.259 |
|  | TSPT | 74 | 0.32 | 0.2 | 0.258 | 0.259 |
|  | Iodent | 67 | 0.298 | 0.15 | 0.239 | 0.229 |
|  | SS | 91 | 0.301 | 0.147 | 0.244 | 0.231 |
|  | P | 63 | 0.332 | 0.184 | 0.268 | 0.261 |
|  | LRC+Lan | 119 | 0.336 | 0.174 | 0.271 | 0.26 |
| K=10 | M-Reid_PA | 55 | 0.315 | 0.184 | 0.254 | 0.251 |
|  | M-Reid_Z58 | 27 | 0.287 | 0.211 | 0.231 | 0.243 |
|  | TSPT_C72 | 46 | 0.302 | 0.214 | 0.244 | 0.253 |
|  | TSPT_HZS | 63 | 0.333 | 0.169 | 0.268 | 0.257 |
|  | IDT | 41 | 0.277 | 0.137 | 0.223 | 0.212 |
|  | X | 29 | 0.278 | 0.161 | 0.224 | 0.221 |
|  | SS | 68 | 0.293 | 0.143 | 0.237 | 0.224 |
|  | P | 40 | 0.316 | 0.19 | 0.255 | 0.254 |
|  | LRC | 34 | 0.299 | 0.18 | 0.241 | 0.24 |
|  | Lan | 87 | 0.332 | 0.167 | 0.237 | 0.245 |

| Table S4 Number of unique , Neutral, and Fixed SNP loci in the SS and Iodent group | | | | |
| --- | --- | --- | --- | --- |
|  |  |  |  |  |
| Chromosome | No.of unique SNP in SS | No.of unique SNP in Iodent | No.of neutral SNP in SS and fixed SNP in Iodent | No.of neutral SNP in Iodent and fixed SNP in SS |
| 1 | 51 | 10 | 2 | 15 |
| 2 | 34 | 12 | 6 | 7 |
| 3 | 38 | 12 | 1 | 5 |
| 4 | 46 | 13 | 7 | 5 |
| 5 | 27 | 6 | 5 | 3 |
| 6 | 24 | 3 | 2 | 4 |
| 7 | 37 | 4 | 2 | 6 |
| 8 | 17 | 4 | 4 | 2 |
| 9 | 20 | 7 | 1 | 1 |
| 10 | 25 | 6 | 2 | 4 |
| Total | 319 | 77 | 32 | 52 |
